# Phylogenetic relatedness can influence cover crop-based weed suppression

**DOI:** 10.1038/s41598-023-43987-x

**Published:** 2023-10-13

**Authors:** Uriel D. Menalled, Richard G. Smith, Stephane Cordeau, Antonio DiTommaso, Sarah J. Pethybridge, Matthew R. Ryan

**Affiliations:** 1 Soil and Crop Sciences Section, School of Integrative Plant Science, Cornell University, Ithaca, NY 14853 USA; 2 Department of Natural Resources and the Environment, University of New Hampshire, Durham, NH 03824 USA; 3 Agroécologie, INRAE, Institut Agro, University Bourgogne, University Bourgogne Franche-Comté, Dijon, France; 4 Pathology and Plant-Microbe Biology Section, School of Integrative Plant Science, Cornell University, Geneva, NY 14456 USA

**Keywords:** Plant ecology, Agroecology, Community ecology

## Abstract

Cover crops are plants grown to provide regulating, supporting, and cultural ecosystem services in managed environments. In agricultural systems, weed suppression services from cover crops can be an important tool to promote sustainability as reliance on herbicides and tillage for weed management has caused pollution, biodiversity loss, and human health issues. However, to effectively use weed suppression services from cover crops, farmers must carefully select species that fit within their rotations and suppress their problematic weeds. Understanding how the relatedness between cover crops and weeds affects their interactions will help farmers select cover crops for targeted weed management. The phylogenetic distance between species reflects their relatedness and was studied through a series of field experiments that compared weed suppression in winter and summer cover crops with tilled controls. This study demonstrates that cover crops can reduce up to 99% of weed biomass and alter weed community structure by suppressing phylogenetically related weed species. Results also suggest that cover crop planting season can influence weed community structure since only overwintering treatments affected the phylogenetic distance of weed communities. In an applied context, these results help develop cover crop-based weed management systems, demonstrating that problematic weeds can be managed by selecting phylogenetically related cover crop species. More broadly, this study provides a framework for evaluating weed communities through a phylogenetic perspective, which provides new insight into plant interactions in agriculture.

## Introduction

Weeds are a major constraint to crop production^1,2^ and are often managed with herbicides and tillage. However, the overuse of both management strategies has contributed to important challenges for agriculture. For example, reliance on herbicides has led to the selection of herbicide-resistant weeds^3^, which reduces the long-term viability of chemical weed management. Of particular concern is increasing weed resistance to multiple herbicide modes of action, threatening the efficacy of existing herbicides^3,4^, and metabolism-based herbicide-resistance pathways, which can compromise the efficacy of future herbicides^4,5^. Although tillage is an effective tool for suppressing herbicide-resistant weeds^6^, reliance on tillage can degrade soil health^7–9^ and result in higher erosion rates than those of natural soil formation^10^. To support crop productivity and food security, it is important to identify weed management strategies that let farmers suppress problematic weeds without degrading agroecosystems.

One approach to sustainable weed management is cover cropping, which is the growth of plants that are primarily intended for regulating natural processes, supporting ecosystem health, and offering cultural benefits to agricultural land rather than solely providing marketable products^11^. When managed properly, cover crops can suppress weeds^12^ while reducing soil erosion^13,14^, improving soil health^13,15^, and diminishing fertilizer requirements in subsequent cash crops^16^. Policymakers recognize these ecosystem services and have shown interest in cover crop usage. For example, in the United States, government programs helped drive a 50% increase in cover crop acreage from 2012 to 2017^11^. Similarly, surveys of farmers in the European Union link government initiatives to increased cover crop use^17^. Research on the effective use of cover crops for weed suppression can help farmers take advantage of an increasingly supportive political environment surrounding cover crop adoption.

Despite clear evidence of weed suppression in properly managed cover crops^12^, there is little consensus on whether cover crops affect weed community structure. Past research highlights this discrepancy because while some experiments report that cover crops affected weed communities^18–20^, others argue that cover cropping had no effect on weed communities^21,22^. Even in experiments where cover crops affected weed community structure, it is unclear if cover crop species or biomass influenced results^18^. The ability to predict how cover cropping affects weed communities would help farmers manage their most problematic weed species, including herbicide-resistant biotypes.

It is important to understand weed community management during the cover crop phase of a crop rotation because these weeds can influence the success of subsequent cash crops. One of the primary ways that weed communities in cover crops affect future crop productivity is through the weed soil seed bank. Soil seed banks are important reservoirs for future weed infestations^23^ and reflect weed suppression of previous crops within a rotation^24–26^. Crops affect the density of weed species within soil seed banks by lowering weed seed production when they reduce weed biomass^27,28^. Consequently, if cover crops change weed community structure by suppressing some weeds more than others, they may influence the weeds in future cash crops^29^.

Patterns in weed community structure could be revealed by examining the phylogenetic relationships of weed species in different crop management scenarios. Phylogenetic trees reflect evolutionary relationships among species based on shared ancestry^30^. The position of a species in a phylogenetic tree reflects its overall ecological grouping^31–36^, particularly when evaluating communities with diverse taxonomic lineages^34^ and comparing communities across habitat gradients^37^. Consequently, evaluating communities with a phylogenetic perspective describes the underlying processes affecting their assembly. Indeed, past research has reported an association between a plant’s phylogenetic grouping and its distribution across different habitat gradients^37–44^. It is possible that the link between plant phylogenetics and habitat tolerance could be relevant in agricultural systems since crop management alters habitat conditions in ways that affect weed community structure^45,46^. Considering the conservation of ecological niches within phylogenetic groups^31–36^, cover crops might create habitats that are more resource-limited to phylogenetically related weed species.

This study examined how cover crops can affect weed community structure by evaluating weed community biomass and phylogenetic distance. We first tested whether the abundance of weed species differed across treatments through a Permutational Multivariate Analysis of Variance (PERMANOVA), an established method of assessing community structure^18,46^. Then, we elaborated upon this approach by testing whether cover crop-based weed suppression affected the phylogenetic distance of weed communities. To test if cover crops had species-specific effects on weed community structure, variation in phylogenetic distance was partitioned by cover crop species and biomass. Finally, to evaluate if cover crops most strongly suppressed related weed species, we tested if cover crops were phylogenetically distinct from coexisting weed communities. The results from this study provide a new approach to weed community research and help farmers create targeted weed management plans.

## Methods

### Site description and experimental design

All experiments in this study were conducted at the Hudson Valley Farm Hub (hereafter referred to as ‘Farm Hub’; Hurley, NY USA 41° 54′ 35.96″, − 74° 5′ 29.41″) and the Cornell University Musgrave research farm (hereafter referred to as ‘Musgrave’; Aurora, NY USA 42° 44′ 2.40″, − 76° 39′ 22.98″). At each site, experiments were repeated over two growing seasons, resulting in four site-years. Within each site-year, all treatments were maintained in 24 by 12 m plots and replicated across four blocks in a randomized complete block design. The weather at both sites is characterized by four distinct seasons, and the Farm Hub and Musgrave sites received an average of 95 and 99 cm of precipitation per year throughout the experiment (2020–2022), respectively^47^.

This study is based on winter and summer cover crop experiments, each comparing four cover crop treatments to a tilled control (n = 80 plots experiment^–1^, total across all four site-years). Winter cover crops are species that are typically planted in the fall and overwinter. The winter experiment evaluated the following cover crops: canola (*Brassica napus*), cereal rye, (*Secale cereale*), hairy vetch (*Vicia villosa*), and a cereal rye × hairy vetch mix. Summer cover crops are planted in spring and grow throughout the summer. The summer experiment evaluated the following cover crops: buckwheat (*Fagopyrum esculentum*), sorghum sudangrass (*Sorghum bicolor* × *Sorghum sudanense*), sunn hemp (*Crotalaria juncea*), and a sorghum sudangrass × sunn hemp mix. All cover crops were seeded using standard practices in our study region (see Supplementary Table 1 for seeding rates, seeding depth, planting date, and equipment used to establish treatments)**.**

Each field used in this experiment had been a uniform crop stand prior to our study. Specifically, before the winter cover crop experiment, spring wheat (*Triticum aestivum*) was grown at the Farm Hub, and oat (*Avena sativa*) and corn (*Zea mays*) were grown at Musgrave before the 2020 and 2021 seasons, respectively. Prior to the summer cover crop experiment, oat and red clover (*Trifolium pratense*) were grown at the Farm Hub in 2020 and 2021, respectively. At the Musgrave site, a cereal rye and hairy vetch mix was grown in 2020, and a spring wheat monoculture was grown in 2021 prior to the summer cover crops.

Before planting the cover crops used for this study, all fields were prepared with tillage. The tilled controls were plots that were not planted with cover crops and reflected the resident weed community that existed in the absence of cover crop-based weed suppression. None of the treatments in this experiment received supplemental fertility, weeding, or irrigation after planting.

### Sampling

Sampling occurred in the standing cover crops, when farmers typically terminate these species to plant their main cash crops. Cover crop maturity at sampling is described using the Biologische, Bundesanstalt, Bundessortenamt, and Chemical (BBCH) scale, a universal descriptor of crop and weed growth^48^. Winter cover crops were sampled in late spring: canola was between BBCH stages 79–81, cereal rye was between BBCH 66–73, and hairy vetch at BBCH 66–75. In the summer experiment, sampling occurred in late summer: buckwheat was between 75 and 82 on a modified BBCH scale^49^, sorghum sudangrass was between BBCH 40–50, and sunn hemp was at BBCH 69–71.

Two 0.25 m^2^ (76 cm × 33 cm) quadrats were randomly placed in each plot to sample crop and weed biomass in each treatment. Each quadrat had a width that was a multiple of the cover crop row spacing and was placed perpendicularly over four crop rows to ensure a consistent proportion of crop row and interrow area in each sample. All individuals larger than 5 cm tall or wide were cut at the soil surface, identified, and stored at the species level. Sampling two quadrats in each experimental unit helped account for variability within cover crop plots. To avoid pseudoreplication, samples were summed at the plot level. The samples were dried at 60 °C for at least 2 weeks before obtaining dry weight for analysis.

### Analysis

Each experiment (winter and summer cover crops) was analyzed separately to ensure that our study compared treatments that had received comparable management. Analyses were based on linear^50^ and generalized^51^ mixed effects models that were carried out using R version 4.2.1^52^. All models accounted for variation through a crossed random intercept that consisted of the four blocks within each site-year. After confirming model fit with the residuals of linear mixed effect models and the simulated residuals^53^ of generalized linear mixed effects models, fixed effects were tested with type three ANOVA tests. Post-hoc tests were then conducted on estimated marginal means and slopes for all categorical and continuous variables, respectively^54^.

To assess cover crop-based weed suppression across the different species, total weed biomass was modeled as a function of the interaction between experimental site and treatment. Post-hoc FisherLSD tests were used to compare weed biomass amongst treatments. Then, to test if cover crop biomass affected weed suppression, weed biomass was described as a function of the interaction between experimental site and cover crop biomass. Weed suppression models in both experiments were generalized linear mixed effects models with a log-linked Tweedie distribution.

PERMANOVA tests were done to determine whether weed community structure differed across treatments in the winter and summer experiments. Before evaluating weed community structure with PERMANOVAs, the homogeneity of multivariate groups was confirmed by assessing beta dispersion of weed communities across all treatment levels. Both PERMANOVA tests evaluated the effect of site, treatment, and their interaction on Bray–Curtis dissimilarity matrixes of natural logarithm plus one transformed weed species biomass. Tests for the two experiments were based on 999 permutations and conducted using the ‘vegan’ package^55^.

All analyses of weed community phylogenetics used abundance-weighted interspecific mean pair-wise distance (interspecific MPD). Compared with other metrics of phylogenetic distance, interspecific MPD has relatively low error rates and is unaffected by species richness^56^. Interspecific MPD was calculated using the phylogenetic variance–covariance matrix of the species in each experiment and weighted using species biomass data. The phylogenetic variance–covariance matrices were made with ‘V.PhyloMaker2’^57^, an R package that creates phylogenetic trees from various molecular-based mega-trees^58^. Across our entire study, five unknown weed species had to be omitted from the phylogenies, and out of the sixty-one remaining species, nine were not defined by ‘V.PhyloMaker2’. All undefined species were replaced by sister taxa identified using published molecular phylogenies (see Supplementary Table 2 for a list of undefined species and the sister taxa used in the analysis). After creating a phylogenetic tree for each experiment using species included in the ‘V.PhyloMaker2’ database, a polytomy across three *Poa* species (*P. annua, P. pratensis*, and *P. trivialis)* was randomly resolved into a series of dichotomies with zero-length branches^59^. Interspecific MPD was calculated with the phylogenetic variance–covariance matrixes of the resulting ultrametric trees.

The interspecific MPD of weed communities was modeled as a function of the interaction between experimental site and treatment to determine if cover crop species affected weed community phylogenetic distance. Post-hoc Fisher’s least significant difference (LSD) tests were used to compare interspecific MPD across treatments and determine whether cover cropping affected weed community phylogenic distance compared with the tilled control. In the winter and summer cover crop experiments, variation in interspecific MPD across treatments was tested with log-linked Gaussian generalized linear mixed effects models. Then, the effect of cover crop biomass on interspecific MPD was tested with linear mixed effects models, where experimental site, cover crop species, and cover crop biomass were interacting fixed effects; because the tilled control had no cover crop biomass, it was excluded from these regressions. The effect of cover crop biomass on interspecific MPD was then compared across treatments with post-hoc Fisher’s LSD tests.

**Figure 1:**
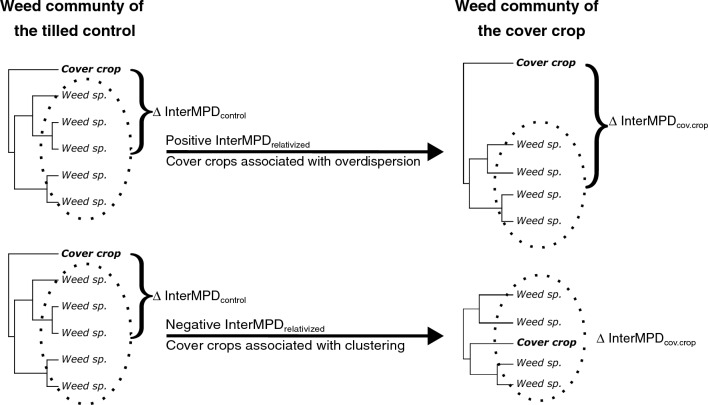
Procedure to evaluate if cover crop-based weed suppression caused treatments to be more or less related to weed communities than expected by chance. Calculating abundance-weighted interspecific mean pair-wise distance (Inter.MPD) with and without the cover crop species (∆Inter.MPD), measures the phylogenetic distance of cover crops relative to weed communities in the cover crop treatments (∆InterMPD_cov.crop_) and the tilled control treatment (∆InterMPD_control_; Eq. (1)). The difference between cover crops and the weed communities in these two treatments (Inter.MPD_relativized_), measures the effect of cover cropping on weed community phylogenetic distance while accounting for any phylogenetic dissimilarity between cover crops and resident weed communities.

After testing if the phylogenetic distance of weed communities in cover crops was different from the tilled controls and describing whether cover crop biomass affected the phylogenetic distance of weed communities, we could determine if cover crop species were more or less related to weed communities than expected by chance (InterMPD_relativized_; Fig. 1). This was accomplished by calculating how phylogenetically distant cover crops were from the weeds in the cover crop treatments (∆InterMPD_cov.crop_) and the tilled control (∆InterMPD_control_; Eq. (1)). Without accounting for the phylogenetic distance between the cover crops and resident weed communities (∆ InterMPD_control_; Eq. (1)), results would be biased by differences between the phylogenetic distance of a cover crop and the weed species in a specific experimental location.

$$\Delta InterMP{D}_{cov. \,\,crop}=InterMP{D}_{cov. \,\,crop + weeds \,\,in\,\, cov. \,\,crop}- InterMP{D}_{weeds \,\,in \,\,cov.\,\, crop,}$$

$$\Delta InterMP{D}_{control}=InterMP{D}_{cov.\,\, crop + weeds \,\,in\,\, control}- InterMP{D}_{weeds\,\, in\,\, control,}$$

1 $$InterMP{D}_{relativized}= \Delta InterMP{D}_{cov. crop}- \Delta InterMP{D}_{control,}$$

For both the winter and summer cover crop experiments, Inter.MPD_relativized_ was assessed as a function of the interaction between experimental sites and cover crop treatments using linear mixed effects models. Post-hoc* t*-tests determined whether positive values indicated overdispersion of weeds relative to the cover crop and negative values phylogenetic clustering of weeds relative to the cover crop.

## Results

**Figure 2:**
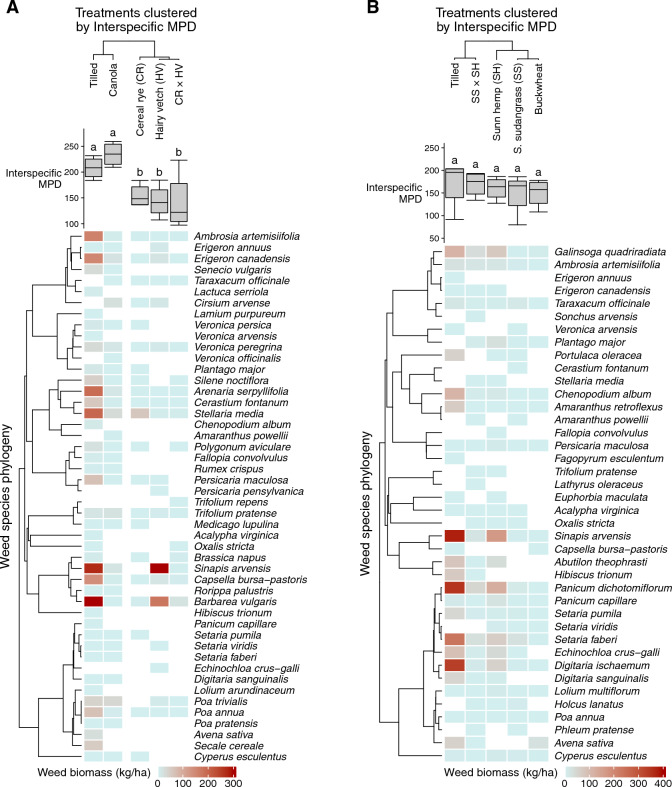
Cover crop effects on weed community phylogenetic distance (boxplots) and biomass (heatmaps) in the winter (**A**) and summer (**B**) cover crop experiments. Treatments are organized through hierarchical clustering, and boxplots show average abundance-weighted interspecific mean pair-wise distance (interspecific MPD). Letters above the boxplots denote the statistical group of each treatment (*P* < 0.05). In both experiments, weed species are arranged using phylogenetic trees. Weed community filtering can be inferred from reduced phylogenetic distance in the boxplots and reduced spread in weed species in the heatmap of the cover-cropped treatments.

The winter and summer cover crop experiments had 48 and 40 weed species, respectively. Samples from the tilled controls reflected the emerged resident weed communities of the two experiments. Average weed biomass in the tilled controls was 2053 kg ha^–1^ in the winter cover crop experiment and 2101 kg ha^–1^ in the summer cover crop experiment. Generally, the tilled controls of the two experiments were dominated by weeds with similar emergence patterns as the cover crops specific to each experiment. For example, the tilled control in the winter cover crop experiment had high amounts of *Barbarea vulgaris*, *Sinapis arvensis*, and *Stellaria media*, which can all emerge in the late summer and fall^60^ (Fig. 2A). Similarly, the control in the summer cover crop experiment had a high biomass of weeds that can emerge in the spring and early summer, such as *Sinapis arvensis*, *Panicum dichotomiflorum*, and *Digitaria ischaemum* (Fig. 2B).

**Figure 3:**
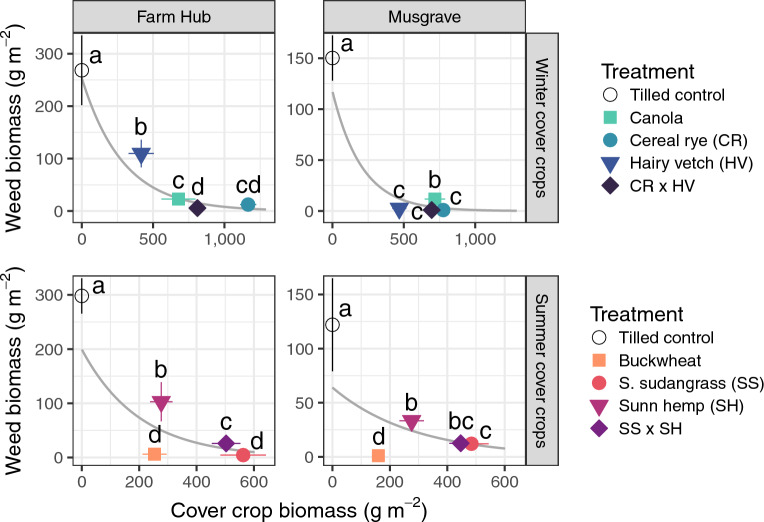
Weed suppression across cover crop biomass and species treatments. Trendlines show the fit of generalized linear mixed effects models with log-linked Tweedie distributions and test whether cover crop biomass affected weed suppression. Letters denote whether weed biomass differed across treatments within each site (*P* < 0.05). Weed biomass is shown across sites to account for the interaction between the experimental site and cover crop treatment (*P* < 0.05).

All cover crop treatments suppressed weeds relative to the tilled control (*P* < 0.05), and both experiments had treatments that suppressed 99% of weed biomass (Fig. 3). This high level of cover crop-based weed suppression was observed in the buckwheat, cereal rye, and cereal rye × hairy vetch mix at Musgrave, and the sorghum sudangrass at Farm Hub. In both experiments, weed suppression increased with cover crop biomass (*P* < 0.001). Furthermore, PERMANOVA tests on weed species biomass indicated that weed community structure differed across treatments in the two experiments (*P* < 0.001).

**Figure 4:**
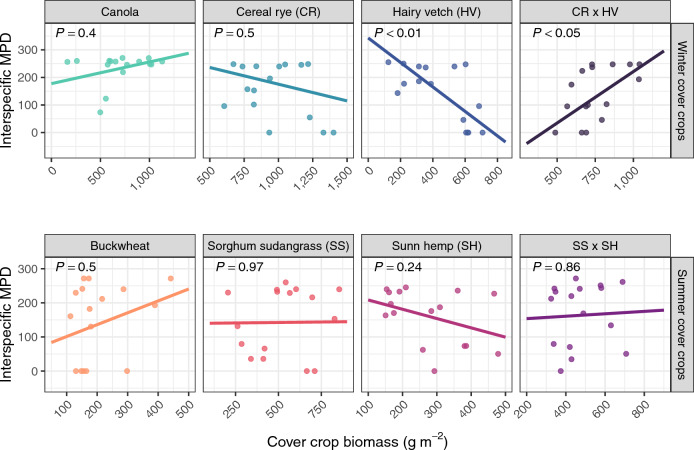
Effects of cover crop biomass on weed community abundance-weighted interspecific mean pair-wise distance (interspecific MPD). The lines in this figure show regression output, and the *P*-values indicate whether the estimated marginal slope of each cover crop is different from zero.

The effect of cover crop species and cover crop biomass on the average phylogenetic distance within weed communities differed across experiments. In the winter cover crop experiment, all treatments except canola reduced weed community interspecific MPD relative to the tilled control (*P* < 0.05; Fig. 2A). However, the extent to which winter cover crop biomass affected interspecific MPD differed by species (*P* < 0.001; Fig. 4). Specifically, canola and cereal rye biomass did not affect interspecific MPD, increasing hairy vetch biomass was associated with a reduction in interspecific MPD (*P* < 0.01), and increasing cereal rye × hairy vetch biomass was associated with increased interspecific MPD (*P* < 0.05). In contrast to the winter experiment, none of the summer cover crops affected weed community interspecific MPD compared with the tilled control (Fig. 2B). Interspecific MPD was also unaffected by the biomass of summer cover crops (*P* = 0.84).

**Figure 5:**
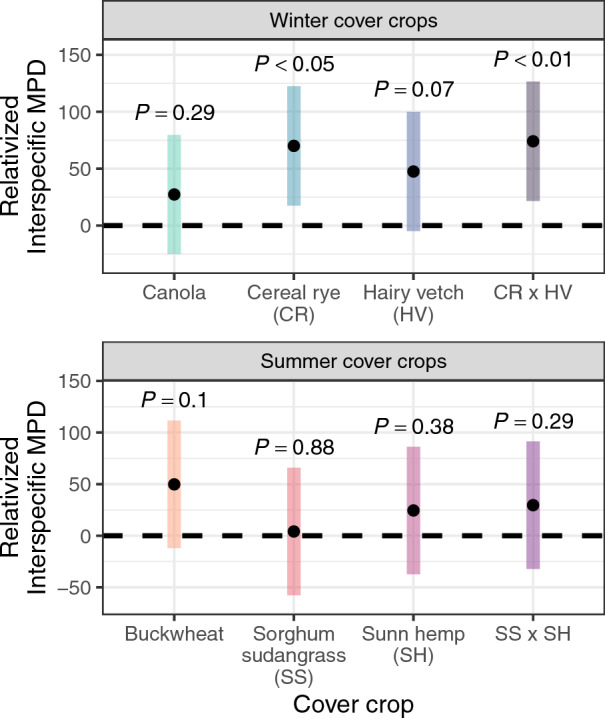
Relativized phylogenetic distance (InterMPD_relativized_; Eq. (1)) between cover crops and weed communities. Points in this figure are estimated marginal means showing the phylogenetic distance between cover crops and the weed communities in the cover crop treatments (∆InterMPD_cov.crop_; Eq. (1)), relativized by the phylogenetic distance caused by cover crop species selection (∆InterMPD_control_; Eq. (1)). Positive values indicate that cover crops were phylogenetically distinct from weeds, whereas negative values indicate weeds were clustered around the cover crop. Error bars are the 95% confidence intervals of the estimated marginal means, and *P*-values indicate whether each marginal mean differed from zero.

Comparing phylogenetic distance between cover crops and their weed communities (∆InterMPD_cov.crop_; Eq. (1)) with cover crops and the weed communities of the tilled control (∆InterMPD_control_; Eq. (1)) describes if phylogenetic relatedness affected weed suppression (Fig. 1). All cover crop treatments with cereal rye were phylogenetically distinct from weed communities (*P* < 0.05; Fig. 5). While the other cover crop treatments were not phylogenetically distinct from weed communities, weed species in these treatments were never more related to cover crops than expected by chance (*P* > 0.05; Fig. 5).

## Discussion

The phylogenetic approach used by this study builds upon past research by providing insight into the underlying ecological structure of weed communities. For example, results from the PERMANOVA—an established analysis of community structure based on species-level biomass—reported that treatments in both the winter and summer experiments affected weed community structure. However, only cover crops in the winter experiment influenced the phylogenetic distance of weed communities (Figs. 2, 4, and 5), indicating that weed suppression in the summer cover crops was independent of the phylogenetic relatedness between cover crops and weeds. Understanding how and why weed suppression was affected by the phylogenetic distance between cover crops and weeds in the winter experiment provides initial steps for targeted weed management with cover crops.

While all cover crops in this study suppressed weeds (Fig. 3), only treatments in the winter experiment changed the phylogenetic distance of weed communities relative to the tilled controls (Fig. 2). Specifically, three of the four cover crop treatments in the winter experiment affected weed community phylogenetic distance: cereal rye, hairy vetch, and cereal rye × hairy vetch. In all cases, phylogenetic distance was lower in weed communities of the cover crops than in the weed communities of the tilled control. The reduction in phylogenetic distance indicates that winter cover crop treatments filtered weed communities by selecting a subset of the possible weed phylogenetic groups.

It is possible that the cover crops in the winter experiment were more likely to influence the phylogenetic distance of weed communities because of their emergence timing relative to summer annual weeds. Unlike the summer cover crops, which emerged alongside summer annual weeds, overwintering cover crops were established by spring, giving them a greater probability of altering the habitat conditions where summer annual weeds had to emerge and establish. The finding that only the biomass of winter cover crops affected phylogenetic distance (Fig. 4) supports this observation, suggesting that the accumulation of cover crop biomass after weed seedling establishment may improve weed suppression (Fig. 3) but not modify the phylogenetic distance of weed communities (Fig. 4).

More broadly, cover crop biomass was examined to determine if the phylogenetic distance of weed communities was influenced by the quantity of cover crop biomass instead of the relatedness between cover crop species and weeds. When cover crop biomass influenced weed community phylogenetic distance in the winter experiment, its effects were inconsistent across species (Fig. 4). Specifically, the reduction in phylogenetic distance compared with the tilled control observed in the cereal rye, hairy vetch, and cereal rye × hairy vetch treatments (Fig. 2A) was only promoted by increased biomass in the hairy vetch treatment (Fig. 4). The inconsistent effect of cover crop biomass on weed community phylogenetic distance aligns with past research, reporting that cover crop biomass was only partially responsible for changes to weed species richness^18^. Thus, cover crops have species-specific effects on weed communities, and species selection may be a means to alter weed community structure.

The effect of phylogenetic relatedness on cover crop-based weed suppression was assessed while accounting for bias that could be introduced by selecting a cover crop species not represented in the resident weed community (Fig. 1). The approach used in this study addresses a knowledge gap in community ecology by evaluating whether plant competition causes phylogenetic clustering or overdispersion at the community level^61^. No cover crop treatment in this study created conditions that selected for phylogenetically related species, and there is no evidence of phylogenetic clustering from crop-weed competition. Instead, weed suppression in two treatments of the winter cover crop experiment, cereal rye and the cereal rye × hairy vetch mix, caused weed communities to be phylogenetically distant from cover crops (Fig. 5), creating phylogenetic overdispersion. Additionally, the hairy vetch treatment, which also affected the phylogenetic distance of weed communities relative to the tilled control (Fig. 2A), was phylogenetically distant from its weeds at marginal significance (*P* = 0.07; Fig. 5). The phylogenetic distance of cover crops in these treatments relative to weed communities indicates that cover crops create conditions that filter weeds by being most suppressive to phylogenetically related species.

## Conclusions

The phylogenetic approach used by this study provides a framework to compare weed communities based on overall ecological similarity. This study provides a preliminary indication that farmers should select cover crops that are phylogenetically related to their most problematic weeds because when cover crops filtered weed communities (Fig. 2), they tended to be most suppressive to related species (Fig. 5). Furthermore, no treatment in this study increased the proportion of weeds that were phylogenetically related to cover crops, so competitive cover crops should not increase the dominance of related weed species. To reinforce this result, future research should compare the phylogenetic distance between weed communities and a broad range of cover crop species.

To improve the probability that cover crops will suppress related weed species, this study suggests that it is important to establish cover crops before weed emergence. Unlike summer cover crops, treatments with overwintering species may have influenced the phylogenetic structure of weed communities because they had relatively more time to modify habitat conditions before summer annual weed emergence. To mechanistically test this finding, future studies could establish cover crops in weed-free conditions and intentionally seed weeds into cover crops at different moments during the growing season. This would directly evaluate whether weed seedling suppression after cover crop establishment determines if phylogenetic relatedness influences weed suppression. The results discussed in this study and the proposed experiments can help farmers select and manage cover crops for targeted weed management. In the face of herbicide resistance and large-scale soil erosion from tillage, using cover crops for weed management will improve the sustainability of crop production and promote long-term food security.

### Supplementary Information

Supplementary Table 1.

Supplementary Information.

## Data Availability

The datasets generated and analysed during the current study are available in the *Cornell eCommons* repository, 10.7298/6gnz-wj06. All data used in this study were collected in accordance with relevant guidelines for plant material collection for field-based research.
