# Supplementary material for: Phylogenetic relatedness can influence cover crop-based weed suppression
Source: Sci Rep. 2023 Oct 13;13:17323. doi: 10.1038/s41598-023-43987-x (PMC10576068; doi:10.1038/s41598-023-43987-x)
Supplement: Supplementary file 1 — Supplementary Table 1. [file 41598_2023_43987_MOESM1_ESM.docx]

**Phylogenetic relatedness can influence cover crop-based weed suppression**

Uriel D. Menalled, Richard G. Smith, Stephane Cordeau, Antonio DiTommaso, Sarah J. Pethybridge, Matthew R. Ryan

| **Crop** | **Site** | **Date** | **Depth (cm)** | **Seeding rate**  **(pure live kg/ha)** | **Seeding Equipment** |
| --- | --- | --- | --- | --- | --- |
| Canola | |  |  |  |  |
|  | Farm Hub | September, 9 2020 | 1.9 | 8 | JD 1590 |
|  |  | September, 14-15 2021 | 1.3 | 8 | JD 1590 |
|  | Musgrave | September, 2-3 2020 | 1.9 | 8 | JD 1590 |
|  |  | September, 7 2021 | 1.3 | 8 | JD 1590 |
| Cereal rye (CR) | |  |  |  |  |
|  | Farm Hub | September, 9 2020 | 3.2 | 210 | JD 1590 |
|  |  | September, 14-15 2021 | 3.8 | 210 | JD 1590 |
|  | Musgrave | September, 2-3 2020 | 3.2 | 210 | JD 1590 |
|  |  | September, 7 2021 | 3.8 | 210 | JD 1590 |
| Hairy vetch (HV) | |  |  |  |  |
|  | Farm Hub | September, 9 2020 | 3.2 | 34 | JD 1590 |
|  |  | September, 14-15 2021 | 3.8 | 34 | JD 1590 |
|  | Musgrave | September, 2-3 2020 | 3.2 | 34 | JD 1590 |
|  |  | September, 7 2021 | 3.8 | 34 | JD 1590 |
| CR × HV | |  |  |  |  |
|  | Farm Hub | September, 9 2020 | 3.2 | 56 (CR) × 34 (HV) | JD 1590 |
|  |  | September, 14-15 2021 | 3.8 | 56 (CR) × 34 (HV) | JD 1590 |
|  | Musgrave | September, 2-3 2020 | 3.2 | 56 (CR) × 34 (HV) | JD 1590 |
|  |  | September, 7 2021 | 3.8 | 56 (CR) × 34 (HV) | JD 1590 |
| Buckwheat | |  |  |  |  |
|  | Farm Hub | Aug, 3 2020 | 2.5 | 56 | JD 1590 |
|  |  | July, 30 2020 | 3 | 56 | JD 1590 |
|  | Musgrave | July, 26 2020 | 2.5 | 56 | JD 1590 |
|  |  | July, 30 2021 | 3 | 56 | JD 1590 |
| Sorghum sudangrass (SS) | | |  |  |  |
|  | Farm Hub | July, 9 2020 | 3.8 | 45 | JD 1590 |
|  |  | July, 16 2021 | 3.8 | 45 | JD 1590 |
|  | Musgrave | July, 7 2020 | 3.8 | 45 | JD 1590 |
|  |  | July, 16 2021 | 3.8 | 45 | JD 1590 |
| Sunn Hemp (SH) | |  |  |  |  |
|  | Farm Hub | July, 9 2020 | 2.5 | 56 | JD 1590 |
|  |  | July, 16 2021 | 3 | 56 | JD 1590 |
|  | Musgrave | July, 7 2020 | 2.5 | 56 | JD 1590 |
|  |  | July, 16 2021 | 2.5 | 56 | JD 1590 |
| SS × SH | |  |  |  |  |
|  | Farm Hub | July, 9 2020 | 2.5 | 16 (SS) × 56 (SH) | JD 1590 |
|  |  | July, 16 2021 | 3 | 16 (SS) × 56 (SH) | JD 1590 |
|  | Musgrave | July, 7 2020 | 2.5 | 16 (SS) × 56 (SH) | JD 1590 |
|  |  | July, 16 2021 | 2.5 | 16 (SS) × 56 (SH) | JD 1590 |

**Supplementary table 1.** Cover crop establishment details. ‘JD’ is short for John Deere.
