# Supplementary material for: Phylogenetic relatedness can influence cover crop-based weed suppression
Source: Sci Rep. 2023 Oct 13;13:17323. doi: 10.1038/s41598-023-43987-x (PMC10576068; doi:10.1038/s41598-023-43987-x)
Supplement: Supplementary file 2 — Supplementary Information. [file 41598_2023_43987_MOESM2_ESM.docx]

**Phylogenetic relatedness can influence cover crop-based weed suppression**

Uriel D. Menalled, Richard G. Smith, Stephane Cordeau, Antonio DiTommaso, Sarah J. Pethybridge, Matthew R. Ryan

| **Undefined species** | **Sister taxa** |
| --- | --- |
| *Acalypha virginica* | *Acalypha rhomboidea*^1^ |
| *Oxalis stricta* | *Oxalis griffithii*^2^ |
| *Sinapis arvensis* | *Brassica nigra*^3^ |
| *Rumex crispus* | *Rheum palmatum*^4,5^ |
| *Sorghum bicolor* × *Sorghum sudanense* | *Sorghum bicolor*^6^ |
| *Lolium arundinaceum* | *Lolium perenne*^7,8^ |
| *Poa annua* | *Cinna spp.*^9^ |
| *Poa pratensis* | *Hyalopoa spp.*^9^ |
| *Poa trivialis* | *Cinna spp.*^9^ |

**Supplementary table 2.** Sampled weed species not in the V.PhyloMaker2 database and their sister taxa, which were defined in V.PhyloMaker2 at the time of publication and used to build the phylogenies in this study.

1. Levin, G. A., Cardinal-McTeague, W. M., Steinmann, V. W. & Sagun, V. G. Phylogeny, Classification, and Character Evolution of Acalypha (Euphorbiaceae: Acalyphoideae). *Systematic Botany* **47**, 477–497 (2022).

2. Deng, T. *et al.* Oxalis wulingensis (Oxalidaceae), an Unusual New Species from Central China. *sbot* **38**, 154–161 (2013).

3. Agerbirk, N., Warwick, S. I., Hansen, P. R. & Olsen, C. E. Sinapis phylogeny and evolution of glucosinolates and specific nitrile degrading enzymes. *Phytochemistry* **69**, 2937–2949 (2008).

4. Grant, K. D. *et al.* A new phylogeny of Rumex (Polygonaceae) adds evolutionary context to the diversity of reproductive systems present in the genus. *PhytoKeys* **204**, 57–72 (2022).

5. Tan, W. *et al.* The complete chloroplast genome of a medicinal resource plant (Rumex crispus). *Mitochondrial DNA B Resour* **4**, 2800–2801.

6. Ananda, G. *et al.* Phylogenetic relationships in the Sorghum genus based on sequencing of the chloroplast and nuclear genes. *The Plant Genome* **14**, e20123 (2021).

7. Cahoon, A. B. *et al.* The complete chloroplast genome of tall fescue (Lolium arundinaceum; Poaceae) and comparison of whole plastomes from the family Poaceae. *Am J Bot* **97**, 49–58 (2010).

8. Torrecilla, P. & Catalán, P. Phylogeny of Broad-Leaved and Fine-Leaved Festuca Lineages (Poaceae) Based on Nuclear ITS Sequences. *Systematic Botany* **27**, 241–251 (2002).

9. Nosov, N. N., Tikhomirov, V. N., Machs, E. M. & Rodionov, A. V. On polyphyly of the former section Ochlopoa and the hybridogenic section Acroleucae (Poa , Poaceae): insights from molecular phylogenetic analyses. *Nordic Journal of Botany* **37**, (2019).
